# Assessing the quality of feedback to general internal medicine residents in a competency-based environment

**Published:** 2019-11-28

**Authors:** Laura Marcotte, Rylan Egan, Eleftherios Soleas, Nancy Dalgarno, Matt Norris, Chris Smith

**Affiliations:** 1 Faculty of Health Sciences, Queen’s University, Ontario, Canada.; 2 Faculty of Education, Queen’s University, Ontario, Canada.

## Abstract

**Construct:**

Competency Based Medical Education (CBME) is designed to use workplace-based assessment (WBA) tools to provide observed assessment and feedback on resident competence. Moreover, WBAs are expected to provide evidence beyond that of more traditional mid- or end-of-rotation assessments [e.g., In Training Evaluation Reports (ITERs)]. In this study, we investigated the quality of feedback in General Internal Medicine (GIM), by comparing WBA and ITER assessment tools.

**Background:**

WBAs are hypothesized to improve written and numerical feedback to support the development and documentation of competence. In this study, we investigated residents’ and preceptors’ perceptions of WBA validity, usability, and reliability and the extent to which WBAs differentiate residents’ performance when compared to ITERs.

**Approach:**

We used a mixed methods approach over a three-year period, including perspectives gathered from focus groups, interviews, along with numerical and narrative comparisons between WBA and ITERs in one GIM program.

**Results:**

Our quantitative analysis of feedback from seven residents’ clinical assessments showed that overall rates of actionable feedback, for both ITERs and WBAs, were low (26%), with only 9% of the total providing an improvement strategy. The provision of quality feedback was not significantly different between tools; although WBAs provided more actionable feedback, ITERs provided more strategies. We found that residents and preceptors indicated the narrative component of feedback was more constructive and effective than numerical scores. Both groups perceived the focus on specific workplace-based feedback was more effective than ITERs.

**Conclusions:**

Participants in this study viewed narrative, actionable, and specific feedback as essential, and an overall preference was found for written feedback over numerical assessments. However, our quantitative analyses showed that specific actionable feedback was rarely documented, despite finding an emphasis from both residents and preceptors of its importance for developing competency. Neither formative WBAs nor summative ITERs clearly provided better feedback, and both may still have a role in overall resident evaluation. Participant views differed in roles and responsibilities, with residents stating that preceptors should be responsible for initiating assessments and vice-versa. These results reveal an incongruence between resident and preceptor perceptions and practice around giving feedback and emphasize opportunities for programs adopting and implementing CBME to address how best to support residents and frontline clinical teachers.

## Abstract (fr)

**Élaboration:**

La formation médicale par compétences (CBME) est conçue pour utiliser les outils d’évaluation en milieu de travail (WBA) afin de fournir une évaluation formative et une rétroaction basés sur l’observationde la compétence des résidents. De plus, les WBA doivent fournir une preuve plus exacte que les évaluations traditionnelles à mi-cycle et en fin de cycle [p. ex. rapports d’évaluation en cours de formation (ITER)]. Dans cette étude, nous avons examiné la qualité de la rétroaction en médecine interne générale (GIM) en comparant les outils d’évaluation WBA et ITER.

**Contexte:**

Les WBA sont pressentis pour être associés à une meilleure rétroaction narrative ou sur échelle quantitative pour appuyer le développement et la documentation de la compétence. Dans cette étude, nous avons examiné les perceptions des résidents et des superviseurs quant à la validité, l’utilité et la fiabilité de la WBA, et la façon dont les WBA différencient les performances des résidents par rapport aux ITER.

**Approche:**

Nous avons utilisé une approche de méthodes mixtes sur une période de trois ans, notamment des perspectives recueillies auprès de groupes de discussion, des entrevues, et également des comparaisons numériques et narratives entre les WBA et les ITER liés à un programme de médecine interne générale.

**Résultats:**

Notre analyse quantitative de rétroaction basée sur sept évaluations cliniques de résidents démontre que les taux globaux de rétroaction pertinente, pour les ITER et les WBA, étaient bas (26 %), et que seulement 9 % de ces deux types d’évaluation suggéraient une stratégie d’amélioration. La qualité de la rétroaction n’était pas très différente entre les outils; les WBA ont fourni plus de rétroaction pertinente, mais les ITER ont fourni plus de stratégies. Selon nos observations, les résidents et les superviseurs ont indiqué que la partie narrative de la rétroaction était plus constructive et efficace que les évaluations par échelles quantitative. Les deux groupes ont estimé que l’accent mis sur la rétroaction en milieu de travail était plus efficace que les ITER.

**Conclusions:**

Les participants à cette étude ont estimé que les rétroactions narratives, pertinentes et spécifiques sont essentielles, et nous avons observé une préférence générale pour la rétroaction narrative plutôt que pour l’évaluation avec échelle quantitative. Cependant, nos analyses quantitatives ont démontré que la rétroaction pertinente spécifique était rarement documentée, bien que les résidents et les superviseurs insistent sur son importance quant au développement des compétences. Ni les WBA formatives ni les ITER sommatifs n’ont clairement fourni de meilleures rétroactions et les deux pourraient toujours avoir un rôle dans l’évaluation globale des résidents. Les opinions des résidents divergent de celles des superviseurs quant aux rôles et les responsabilités : les résidents affirment que les précepteurs ont la responsabilité d’initier les évaluations, et vice versa. Ces résultats révèlent une discordance entre les perceptions et les pratiques des résidents et des superviseurs quant aux rétroactions à apporter. Ils mettent également l’accent sur les opportunités pouraux les programmes qui adoptent et implantent la formation médicale par compétences pour trouver la meilleure façon d’appuyer les résidents et les enseignants cliniques sur le terrain.

## Introduction

In 2015, the Royal College of Physicians and Surgeons of Canada (RCPSC) announced the implementation of a Competency by Design (CBD) initiative that was developed within a competency-based medical education (CBME) approach.^1^ Although CBME is a relatively new initiative in Canada for Royal College Specialties, the College of Family Physicians of Canada and other nations have been engaged in this approach to residency education for a number of years.^2–5^ Findings across these settings indicate that while the idea of CBME is popular amongst trainees and assessors, it is not without challenges, including residents being resistant to increased observation and feedback,^6–9^ and preceptor concerns that CBME will be more time-consuming and onerous.^10–13^

The goal of assessment in medical training is twofold. First, if it is delivered properly, assessment drives the learning process.^14,15^ Second, it also provides important documentation of performance and overall resident competence, regardless of whether a program is structured in a traditional time-based format, or in a competency-based format. With the global shift towards CBME, there has been a parallel shift towards implementing workplace-based assessment (WBA).^11,16^ WBA refers to frequent, formative, and criterion-referenced clinical assessment.^17,18^ Grounded in adult learning principles^19–22^, a CBME approach using WBAs promotes self-directed learning early in residents’ medical training.^17,23,24^ These formative assessments also offer timely opportunities for preceptors and academic advisors to identify and coach residents who are in difficulty. The CBD framework must provide sufficient competency data to validly and reliably assess resident competence. Determining which types of assessment tools will be most effective, and how best to implement them, remains a challenge in medical education.^9,11^ WBAs are believed to improve timeliness and specificity of assessment and to prompt actionable feedback at the time of a clinical encounter.

Feedback is considered effective if it fosters ongoing resident learning through these qualities descriptive, narrative, task-focused, specific, criterion-based, timely, constructive and actionable.^17,25^ Constructive and actionable feedback are described as providing direction for improvement including identifying a specific area or strategy for action^25^. This type of feedback attributes residents’ performance to controllable behaviours that allows residents to progress towards a learning goal^26–29^. This type of quality feedback is especially important in a CBME environment where learning is more resident- centred and preceptors are “expected to directly observe trainees and provide context-specific, behaviorally based feedback to learners”.^30^ There is a gap in knowledge about whether formative WBA provide improved documented feedback as compared to traditional summative ITERs.

The purpose of this study was to determine whether, within one postgraduate medical subspecialty training program, there was a difference in the quality of feedback between summative ITER assessments that characterize a pre-CBME environment and the formative WBAs that will characterize the CBME model. Our research questions included,

1. How does the perceived and assessed quality of feedback differ with the implementation of the WBAs?
2. To what extent were the assessment tools perceived to be usable, valuable and feasible?
3. In what ways did WBAs document the development of a resident’s competence?

## Context

The General Internal Medicine (GIM) program at Queen’s is a two-year medicine subspecialty program of PGY-4 and -5 trainees. GIM began preparing for the implementation of CBD assessment processes in 2015 by designing and implementing several rubric-based assessment tools. Rubrics assessed between 1-15 skills (if observed), across seven CanMEDS Roles.^31^ Traditional ITERs were updated but continued to be used as WBAs were introduced. Appendices A and B provide examples of a representative ITER and WBA tools, respectively.

GIM preceptors and residents received background information on CBME and workplace based assessment, as well as training on when and how to complete the new forms. Emphasis was placed on providing constructive, narrative feedback on any assessment form. Regular email reminders were sent to complete WBA in certain clinical environments (e.g. longitudinal GIM clinic). Each resident was assigned an academic advisor (who at the time of this study was the program director), who met with the resident at three month intervals to review the assessment portfolio, summarize progress, and provide longitudinal coaching, in the model originally suggested by the RCPSC.

## Methods

We used a mixed methods analysis with a concurrent triangulation design in this study.^32,33^ We supported assessment effectiveness by a retrospective quantitative analysis of assessment scores with a qualitative analysis of preceptor narrative assessment feedback spanning four years (2013-2016). We juxtaposed narrative data from interviews and focus groups with assessment data to provide a rationale for evaluation outcomes and to recommend future improvements to residency assessment.

### Setting and participants

GIM at Queen’s is a mid-sized medical subspecialty training program with seven PGY-4/5 residents and 11 GIM preceptors at the time of this study (2015/2016). The residents performed rotations in a wide variety of medical contexts, many with non-GIM rotations and assessors (e.g., stress testing with a cardiologist, or a radiology elective). Assessment data was collected across a number of contexts from residents, and from a variety of GIM and non-GIM preceptors. We conducted this research between January 2016 and January 2017 in the lead up to formal CBME implementation. Ethical approval was provided by the Queen’s University Health Sciences Research Ethics Board.

### Data collection

Both qualitative and quantitative data were collected. For the qualitative data, participation by both residents and preceptors was voluntary. Quantitative assessment data were collected prior to conducting the interviews and focus groups to ensure assessments were not influenced by interview and focus group discussions.

Using convenience sampling, resident interviews were conducted with four of the seven residents who agreed to participate in the study (two PGY-4s and two PGY-5s). The PGY-4s had been exposed to both WBAs and ITERs whereas the PGY-5s had been exposed to mainly ITERs in their PGY-4 year, and to both ITERs and WBAs in their PGY-5 year. The interviews were between 30-55 minutes in length.

Seven of nine GIM preceptors (78%) agreed to participate in two focus groups (n=2; n=5), one before and one following the resident interviews. Residents and preceptors participated separately in the study to ensure both groups felt able to speak freely and critically. Focus groups were 60 minutes in duration.

Expert research interviewers who were not associated with the GIM training program and had extensive experience with qualitative research conducted the interviews and focus groups. One researcher external to the department conducted all the interviews, while a second external researcher conducted the focus groups. Both external researchers attended the focus groups, one conducted the focus groups and the second took notes, identified preliminary themes, and summarize findings for participant verification. Guided by the literature, the research team developed the focus group guide (protocol) and revised the questions based on the results of the first interview.

The protocols targeted three key areas including, 1) residents’ and faculty perspectives on requirements for quality feedback, 2) differences between formal and informal feedback, and 3) the perceived usability, feasibility, and value of the WBA tools (see Appendix C for protocols). All interviews and focus groups were audio recorded and transcribed verbatim. Pseudonyms replaced identifiable information to protect participant confidentiality.

Quantitative data consisted of numerical resident assessment data and were collected before conducting the interviews and focus groups to ensure feedback forms completed were not influenced by the interview and focus group discussions. These quantitative data were organized into spreadsheets and data cleaned to enable statistical analyses. We quantified all written feedback from the resident assessment tools as not-actionable, actionable, or actionable with strategy. If the feedback described the competency of a specific task it was coded as “actionable”, if a specific strategy for improving the task was provided it was coded as “actionable with a strategy”. If the behaviour or skill was not identified it was considered not-actionable (e.g. “great work”). Actionable with strategy was considered more beneficial (see introduction) as it assists residents’ ability to self-regulate their learning.^34–38^

### Data analysis

Our analysis began by entering the data for all assessments completed between 2014 and 2016 into our statistical software (SPSS v21) for descriptive and inferential statistical exploration and testing. After data was inspected for outliers, typos, and normality (ANOVAs) inferential statistical analysis was conducted. Chi-Squared tests were used to test if there were statistically different feedback provided across assessors and assessment categories as a means of showing differences in quality or other factors. Phi coefficients (φ*_c_*) were calculated to determine the level of association between assessors and the quality of feedback provided. A Factorial ANOVA was used to test for differences between assessors and their average scores and partial-eta- squared effect sizes (η^2^) were calculated to demonstrate magnitude of difference.

We conducted qualitative analysis using a thematic design after all data were collected as a means of identifying perceptions about the current assessments and process, and strategies for future improvement.^39^ Using open coding, two researchers independently coded one interview and one focus group to ensure inter-rater reliability and formulate the codebook (91.35% of coding was the same and 8.65% of codes were changed with consensus for the reliability of meaning). We merged the 473 codes across all the interviews and focus groups into 28 distinct super-codes, 13 categories, and 4 themes through research team meetings until consensus was reached. We triangulated all data to ensure a connection between both the quantitative and qualitative results. The quantitative results are embedded into each theme in the results section when there is a direct relationship to the qualitative data.

## Results

**Figure 1:**
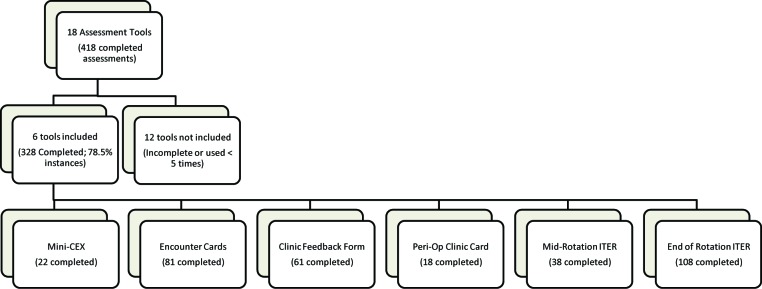
Study inclusion and exclusion criteria of assessment tools

Figure 1 depicts the number of assessment tools included and excluded in this study. The 11 GIM preceptors performed 55% of these assessments (180/328), whereas 57 non-GIM preceptors performed the remaining 45% of assessments (148/328).

We found that four themes emerged from the mixed method analyses: (1) Desiring targeted, formative feedback; (2) Addressing usability, reliability and value of assessment tools; (3) Identifying who is responsible for initiating assessments; and (4) Synthesizing summative and formative feedback to assess resident competence. Quantitative data are included within each of the themes where there is a specific link in order to provide additional evidence that supports the findings. Tables 1, 3-5 provide the topics within each theme with selected quotations.

**Table 1:** Occurrence of actionable feedback

Tool		Feedback	
	Written Feedback	***Actionable*** “I have encouraged him to continue reading around ECG and stress test interpretation to achieve mastery of the subject.”	***Actionable (with a Strategy)*** “Subscribe to [a journal] watch application to know what is going on in the field”
WBA	132/182 (71%)	46/182 (25%)	19/182 (10%)
ITER	111/146 (0.76)	38/146 (26%)	17/146 (12%)
Total	243/328 (74%)	84/328 (26%); *p*> 0.1	36/328 (11%); *p*> 0.1

***Theme 1: Desiring targeted, formative feedback*.** As pertinent to research question 1,**** participants expressed a desire for targeted, narrative, formative and constructive feedback.**** ITERs were seen by both preceptors and residents as having minimal effectiveness with the exception of the comments section which was seen as valuable. Residents were unanimous in the belief that the narrative component of the ITERs was more effective than scores on a numerical scale for providing constructive feedback. The WBAs were viewed as more useful as they were more likely to provide information about resident progression through the training program. Both residents and preceptors believed the focus on written comments through the WBAs was a more effective assessment. Most participants noted that WBAs were more effective than ITERs at providing feedback based on the resident level, timeliness and specificity.

All residents distinguished between the formal and informal feedback that they received. Informal feedback was preferred; it was timelier and tended to occur soon after the direct observation, whereas formal feedback was viewed as less timely. The majority of participants described the logistical challenges of written feedback in a busy clinical environment. Both residents and preceptors identified the importance of an academic advisor in providing residents with targeted, actionable feedback.

There was a contradiction, however, between the perceived actionability and specificity of WBA assessment feedback, and non-significant statistical differences between the two types of assessment. Across the six high frequency tools with identifiable assessors, preceptors provided written feedback 74% of the time (243/328). The extent of feedback was not differentiated by tool type as both ITERs and WBAs returned similar amounts of feedback that was actionable, and actionable with a strategy, as indicated by a Chi-Square test (*χ*^2^ = 2.69, *p* = .261). As well, there is no relationship between the numerical scores received on assessments and actionable feedback as all the residents scored high consistently indicating that none of the tools were discriminatory (Table 1).

Twenty-one of 72 physicians (29%) accounted for the provision of all actionable feedback and 68% (222/328) of all assessments completed. Of the top 10 most frequent preceptors, nine were core GIM faculty while the 10^th^ was a chief preceptor of a community- based rotation. Twenty-one percent of the total number of assessors were GIM faculty and they contributed to 55% of the six assessment tools used in this study. There was a significant association between assessors and the provision of actionable and actionable with strategy feedback (*χ*^2^=41.22, df=18, p<.001, and φ*_c_*=.318 and *p*<.001). To determine if score ranges differed significantly across the 10 high frequency assessors, an ANOVA was used to determine the relationship between assessors and average scores. There was a significant difference between assessors and average scores (*F*(9, 213)=12.49, *p*<.0001, η^2^=.345) which is commonly understood to constitute a large effect size. Among the top 10 assessors, there was an uneven distribution of assessments per resident. For example, Resident-3 had 12/28 assessments provided by Preceptor-23 and none provided by Preceptor-9 despite the fact that Preceptor-9 provided the highest number of assessments to residents overall. Given the large number of assessments performed by Preceptor-9, it is extremely unlikely this avoidance was random.

**Table 2:** Theme 1 selected quotations

Theme 1 Selected Quotations
*The only useful thing in the ITERs was the comment section…. Whereas, the WBAs will create a small movie for you on how you are progressing. It actually gives you direction on how to improve the skills sets that you are lacking so you become better at being a physician…. The informal assessments are more useful than the formal ones during the ITERs. (I-Bryan)* *If the attending or the evaluator filled out the comments [on the ITERs] that would be useful for me tochange something or to improve on it. (I-Dalia)* *The one thing that I like about ITERs is…the narrative component at the end. When you have time to reflect on a few clinics or a few experiences with someone, you can get slightlydifferent perspective and information…. [WBAs are] really data moments for the bigger conversation…. You need time. I observe one and I lose track of the other seven.. (FG1)* *I’ve found that the feedback doesn’t really make all that much sense until someone else pulls it all together…. Sometimes you get that five second feedback but then you have to run off…. It is just not practical to be quite honest. (I-Allison)* *It really takes that overarching person to [state that], ‘On the last four rotations people have commented that your communication skills are a challenge, so why don’t you work on that going forward?’. (FG2)* *Going forward, they have identified areas which are for our level [about] where we should be aiming to improve our knowledge or our practice in the future. It isn’t necessarily areas of deficiency but expected areas of improvement for everyone going forward. (I–Fiona)*

***Theme 2: Addressing usability, reliability and value*** ***in assessment tools*.** In addressing research question 2, resident and preceptor needs for improving the quality of feedback related in part to the usability, reliability, and value of the assessment tools. Within these dimensions preceptors noted the importance of understanding how to incorporate numerous points of feedback from multiple sources and to overcome barriers associated with reacting to constructive feedback.

Residents believed that assessors rushed to complete narrative feedback since it was always the last section on WBAs. To overcome this challenge, many participants suggested that providing prompts in the ‘comments’ section of the tools, would increase the feasibility and usability of assessment forms. Preceptors also stated that, at times, they required additional time to write thoughtful feedback, rather than attempting to write quality comments in-the- moment. Many participants believed that given the current clinical environment, it was not always feasible to give timely feedback in an appropriate setting, and may decrease the reliability of comments between assessors.

All participants were more approving of the new WBAs and their potential to improve useful feedback.

Differentiation based on written feedback was shown to be essential for overall assessment and coaching of residents due to the lack of performance differentiation and ceiling effects found across numerical assessment scores. For ITER’s, 100% of Mid-Rotation assessments and 71% of End-of- Rotation ITERs received a perfect score. WBAs received a weighted average score of 87.5% with a total of 75 perfect scored (41% of the total WBAs). ITERs received a weighted average of 86.8% with a total of 44 perfect scored (30% of total ITERs). A comparison on the average scores of the seven CanMEDS roles resulted in no significant difference between tools.

**Table 3:** Theme 2 selected quotations

Theme 2 Selected Quotations
*You have to make it [assessment tool] short and sweet. At most there should be three little things [that] someone cancheck off. The rest…[they] have to write a comment. Any generalized comments [are] not helpful. I would say, ‘Today was there anything in the clinicalsituation that they could have changed their decision making?’ (I-Allison)* *I think what I struggle with is that I usually want to put a bit more thought into the feedback. (FG2)* *I don’t think it is feasible [to complete an assessment] every time. I don’t think that is possible…with the current model of work that we are doing…. I have had instances with residents where I am giving them what I feel is very constructive feedback. They are so offended by the conversation because their expectation was differentthat Idon’t actually feel that they are actually benefiting from what I am saying to them. (FG1)* *The WBA’s have the potential of giving you directed on-site feedback as long as you get observed…. That is the biggest factor involved in making sure that the feedback is actually useful. (I-Bryan)* *I would prefer the process the way it is now where we get more observation and it is based more on tasks and more regular assessments. (I-Fiona)* *I thought that was a nice way of having feedback that was not just onesnapshot…. It was more, ‘Overall, here is where you are at’…. I still find it difficult [to receive constructive feedback] because I take it personally or as criticism. (I-Dalia)*

***Theme 3:*** **Identifying who is responsible for initiating assessments.** Aligned with research question 2, the need to explicitly identify the roles and responsibilities for the resident assessments emerged from the data and included identifying responsibility for initiating assessments and the willingness of some assessors to complete the WBAs. Most residents believed that the preceptor should be responsible for initiating the assessments. Residents reported feeling uncomfortable initiating the feedback process due to the possibility of a negative assessment and not knowing the assessor during short rotations. Preceptors agreed that narrative feedback was important, however, in contrast to the resident perspective, they suggested that the residents should be the initiators. They felt that this was currently not the case. Although, some preceptors showed concern that full autonomy would result in residents targeting assessors who provide only positive feedback.

Most residents felt that the willingness to provide feedback differed greatly between preceptors. They suggested that the more interested a preceptor was in the teaching and learning process, the better they were at providing effective feedback. Both residents and preceptors agreed that there was a need to improve the quality of feedback. Finally, participants described the importance of receiving multiple sources of feedback—not only from preceptors but also from allied health professionals, which in turn, provides a more complete assessment of their strengths and weaknesses.

**Table 4:** Theme 3 selected quotations

Theme 3 Selected Quotations
*I am scared of getting bad feedback…I find it hard to ask forfeedback…the 360 perspective. (I-Fiona)* *But that was mostly attending based. I did not get any feedback from patients or Allied Health services or other people…. Some preceptors were interested [in providing feedback] and other were not…. I think it is just how much they are involved in the teaching or involved in the learning…. I find that some of the ITERS…are very vague…. They will just write little things about you but it is very vague. (I-Bryan)* *“In my experience [it is] not very frequently that a resident will approach me and ask for some specific feedback for a specific episode…. I think one of the challenges is going to be if the model becomes resident-driven… They will pull out the card [WBA] when they have done a good job. You are not going to say, ‘I performed this code abysmally can you evaluate my performance?’ (FG2)*

***Theme 4: Synthesizing summative and formative feedback to assess resident competency development*.** As pertinent to research question 3, the results demonstrate the importance of incorporating both point of care and longitudinal feedback when making decisions about a resident’s competence. Most participants agreed that longitudinal feedback was a valuable component of a robust assessment portfolio. The ITERs and WBAs together, were viewed as a more complete method of assessment than the ITERs alone. Many participants were satisfied that the WBAs could provide support for the ITER data. They felt that the WBAs bridged the practice-feedback latency gap caused by ITERs. The ITERs allowed preceptors to comment on trends of performance, whereas WBAs provide data points demonstrating an overall pattern en route to a resident’s development of competence.

**Table 5:** Theme 4 selected quotations

Theme 4 Selected Quotations
*I feel like each of them, on their own, has pros and cons. Ideally, there would be some sort of blend of the two whereyou would get the whole picture. (I-Dalia)* *I think each has its own advantages, ITERS do somethings well and WBAs do other things, you’d need them both. (FG2)*

## Discussion

In line with finding from contemporary literature, our qualitative data described the importance of timely, high quality feedback, and the potential for WBAs to be more effective than ITERs in this regard. ^40,41^ However, our quantitative results showed there was no difference in the quality of feedback documented between the two methods. The scores residents received on both assessments were near the highest of the scale with little standard deviation, and are unlikely to differentiate between resident capabilities or provide meaningful feedback to the trainees on areas to target for improvement. This was true whether traditional scales (does not meet/meets/exceeds expectations) in an ITER format, or a three-point scale using behavioural anchors (not yet/almost/achieves) in a WBA rubric format were used. This corresponds to findings from the participants, who felt that numerical scores did not provide useful feedback whereas the written comments were the deepest source of value. Qualitatively, one might expect WBAs to provide narrative snapshots of a resident’s performance, with granular bits of specific formative feedback based on the case at hand or the experience of the day. In fact, our results demonstrated no difference in the frequency or quality of actionable feedback provided to residents, using traditional ITERs versus newer WBAs.

This lack of difference between assessment tool types may imply that the provision of high-quality feedback has more to do with preceptor development and the culture of assessment and feedback, than with the assessment tools themselves. Both residents and preceptors in this study expressed a desire for guidance in how to best implement and operationalize feedback strategies. Traditional thinking in regard to fostering a culture of feedback has focused on preceptor and resident development to improve the giving and receiving of feedback,^10^ but Harrison and colleagues offer novel ideas around structuring programmatic assessment to improve students’ receptivity to feedback, including themes of emphasizing trainee personal agency or autonomy, and maintaining authenticity and relevance to practice environment when developing assessments.^42^ These factors should be taken into account when developing assessment tools and strategies for implementation. Literature shows the negative influence that summative assessments can have on receptivity to feedback and this will require close consideration moving competency-based assessments forward^42,43^

Our results showed that providing timely, constructive and actionable feedback in a safe environment was also seen as essential in a CBME culture, although this type of feedback was rarely given. Administrative support in terms of creating schedules that allowed for completing WBAs, and preceptor development on what constitutes quality feedback will be important if the culture of assessment is to truly shift. Finally, the academic advisor system for collating assessments and interpreting results in terms of competency development was seen as valuable for resident understanding of their learning.

Our data also reveal a disconnect between residents and preceptors regarding the responsibility for initiating WBAs. Each group thought the other should be the one primarily responsible for initiating feedback and completing the WBAs. This is an important issue to recognize and address, as it is frequently proposed that CBME will be a learner- driven process.^44,45^ Preceptors in our study suggested the need for a schedule such that residents do not ‘cherry pick’ their assessors, while also ensuring preceptors complete a required number of assessments for the residents they supervise. Our research showed an uneven distribution of completed assessments between some residents and preceptors. This may be due to the scheduling of resident rotations which does not always ensure equal pairing of residents and assessors, but certainly raises the possibility of cherry picking.

Finally, the results show a discrepancy between preceptors who consistently provide high quality feedback and those who do not. Both residents and preceptors clearly articulated the value of an academic advisor, or long-term mentor, in helping them translate feedback from assessments into learning and personal development. This person plays a critical role in helping residents see patterns of feedback, putting formative assessment in an overall context, and ultimately helping them reflect, accept and act on feedback.

Our results suggest a need for further professional development with preceptors, academic advisors, and residents, on both the process for using WBA tools, and on how to effectively deliver and interpret actionable feedback from a spectrum of evaluations.

### Limitations

This study took place in a single division at one hospital site with a small sample size. Therefore, generalizability of our findings to other contexts should be made with caution. We feel that the emergent themes from our study are important and relevant, however it is certainly possible that additional themes would emerge by replicating this study across specialties. Faculty training within this single division could also be improved given the evidence provided that the tool used mattered less than the assessment or feedback skill of faculty.

### Conclusion

Constructive, formative, narrative feedback is an important element in medical training programs, both to drive trainees’ learning and to document resident progress and competence, as recognized by both preceptors and residents. WBAs are regarded highly as a tool for narrative and more frequent feedback. However, in our study of WBA compared to traditional ITER assessments, there was no difference in the documentation of specific actionable feedback, which was low in both assessment types. This demonstrates that simply creating WBA tools does not necessarily translate into more explicit or actionable feedback for trainees, an important concept to consider as many medical training programs begin the transition to CBME. This may reflect the need for preceptor development, and addressing the culture of learning and feedback which is independent of assessment tools.

### Practice points

- Targeted, narrative, formative and constructive feedback is desired for CBME.
- Address both preceptor and resident needs if the goals of competency-based assessments are to be reached.
- Identify roles and responsibilities of those charged with completing competency-based assessments if the competency-based assessment system is to be effective.
- A process for synthesizing both formative and summative resident feedback is needed for making competency development decisions.
- Successful integration of CBME takes thoughtful systemic development and dedication at all levels of the adoption and implementation process.
- It is important for residents not to cherry pick their preceptor to skew their WBAs.

## Appendix A - An In-Training Evaluation Record (ITER)

Assessors were asked to comment on a resident’s competence after a rotation by filling out the below assessment form. There was an additional space for a narrative description or qualitative comments.

**Considering how the fellow’s performance met (or did not meet) the statements above, please take the time to provide some narrative feedback. *This is the most important part of the ITER*. All feedback should be viewed as formative and constructive, and only one part of a global, multimodal system of assessment**.

Please comment on areas of strength; this may be general feedback, or based on particular patient encounters or areas where the fellow demonstrated particular skill:

Please comment on areas for potential growth; this may be general feedback, or based on particular patient encounters or observed patterns where the fellow could improve:

## Appendix B - An example of one of the WBA tools (Encounter Card)

Assessors were asked to complete the following encounter card directly after observing the resident in a clinical setting on a specific case.

## Appendix C: Interview and focus group protocol

**General Internal Medicine Assessment Interview/Focus Group Protocol**

**[This version was for Faculty, word substitutions of Preceptors to Residents were made when interviewing Residents]**

**Please take a moment to read and sign the consent form**.

***Dimension 1: General satisfaction with feedback***

**What is the most common way you provide feedback to residents?**

What other types of feedback do you provide?

Does the type of feedback you provide differ for residents at different stages of their training?

**Do you think the residents find the feedback they receive effective?**

Is formal or informal feedback more effective? Why?

**What makes feedback effective?**

[Probes: Consider asking about Specific, Measurable, Attainable, Relevant, and Time Bound]

[Consider] What makes feedback effective? (Probes: timely, feasible, informative, constructive)

Dimension 2: Attitudes about feedback

**When feedback is effective how do you think it makes residents feel?**

**From your perspective, what makes feedback ineffective?**

When you have provided ineffective feedback, how do you think it made residents feel?

Do you think it’s possible to always give and receive effective feedback? Why or why not?

Do you think residents are in a position where they feel they can ask for specific feedback on a given task or skillset?

Is it typically feasible to provide specific? Can you provide an example?

What might put you in a better position to provide residents with feedback?

To what extent do ITERs and WBAs enable directed feedback?

**Do residents think that** ***you intend*** **your feedback to be coaching or criticism?**

**Do you think** ***residents perceive*** **the feedback you provide as coaching or criticism?**

Is there a difference between the ITERs and WBAs in terms of whether feedback comes across as coaching or criticism?

Please provide an example

**Please consider both the ITERs and the WBAs. Which one do you think is more helpful?**

Why do you think this from your perspective?

Was feedback from one tool more feasible than the other? If so, how?

**Do you think ITERs and WBAs are fundamentally different?**

How would you describe any differences?

***Dimension 3: Applicability of feedback***

**To what extent is feedback provided by the WBA or ITERs more timely?**

What are the barriers to providing timely feedback?

Is there a way to make feedback more timely?

**Please describe how feedback provided by ITERs and/or WBA allow residents to set learning goals, and determine if you have obtained them?**

Do these tools help residents identify areas where there is opportunity for growth?

Do they help residents determine areas for improvement in the future?

**In what ways do you feel residents would be able to** ***advocate for their progression*** **through the residency program based on feedback from WBA and ITERs, if at all?**

Would one assessment be more useful than the other at enabling residents to advocate for progression through the program?

**Have you found that the feedback from the WBA and ITERs is specific to areas of residents’ concern? If so, what areas of concern?**

Can you provide a specific example when this was the case?

Is the feedback residents receive from WBA and ITERs specific to their personal performance?

Is there a difference between the two tools?

**Do you feel that feedback provided from WBA and ITERs is sufficient to highlight areas of strength?**

**Do you feel that feedback provided from WBA and ITERs is sufficient to highlight areas where improvement is needed?**

Is it personalized, or more generic?

Do you think personalization for each person is feasible? Why or why not?
